# Assessing Attitudes towards Statistics among Medical Students: Psychometric Properties of the Serbian Version of the Survey of Attitudes Towards Statistics (SATS)

**DOI:** 10.1371/journal.pone.0112567

**Published:** 2014-11-18

**Authors:** Dejana Stanisavljevic, Goran Trajkovic, Jelena Marinkovic, Zoran Bukumiric, Andja Cirkovic, Natasa Milic

**Affiliations:** Institute for Medical Statistics and Informatics, Faculty of Medicine, University of Belgrade, Serbia; Tilburg University, Netherlands

## Abstract

**Background:**

Medical statistics has become important and relevant for future doctors, enabling them to practice evidence based medicine. Recent studies report that students’ attitudes towards statistics play an important role in their statistics achievements. The aim of the study was to test the psychometric properties of the Serbian version of the Survey of Attitudes Towards Statistics (SATS) in order to acquire a valid instrument to measure attitudes inside the Serbian educational context.

**Methods:**

The validation study was performed on a cohort of 417 medical students who were enrolled in an obligatory introductory statistics course. The SATS adaptation was based on an internationally accepted methodology for translation and cultural adaptation. Psychometric properties of the Serbian version of the SATS were analyzed through the examination of factorial structure and internal consistency.

**Results:**

Most medical students held positive attitudes towards statistics. The average total SATS score was above neutral (4.3±0.8), and varied from 1.9 to 6.2. Confirmatory factor analysis validated the six-factor structure of the questionnaire (Affect, Cognitive Competence, Value, Difficulty, Interest and Effort). Values for fit indices TLI (0.940) and CFI (0.961) were above the cut-off of ≥0.90. The RMSEA value of 0.064 (0.051–0.078) was below the suggested value of ≤0.08. Cronbach’s alpha of the entire scale was 0.90, indicating scale reliability. In a multivariate regression model, self-rating of ability in mathematics and current grade point average were significantly associated with the total SATS score after adjusting for age and gender.

**Conclusion:**

Present study provided the evidence for the appropriate metric properties of the Serbian version of SATS. Confirmatory factor analysis validated the six-factor structure of the scale. The SATS might be reliable and a valid instrument for identifying medical students’ attitudes towards statistics in the Serbian educational context.

## Background

Medical statistics has become important and relevant for future doctors, enabling them to practice evidence based medicine. It is impossible to develop the skills needed for the critical evaluation of evidence in published medical literature and complex decision making in everyday clinical practice without at least a basic knowledge of statistics [1]–[5].

In Serbia, an introductory course in medical statistics is compulsory for third year medical students. The syllabus includes descriptive statistics, some major probability distributions, parameter estimation, hypothesis testing, correlation and regression, and the application of various statistical software solutions. This introductory course is important, as it often is the only statistics course that medical doctors undertake.

In the past, attention was paid predominantly to improving the cognitive aspects of statistics instruction, while more recently, research in statistics education has focused on the assessment and roles of non-cognitive aspects, such as students’ attitudes towards statistics [2], [6]. Attitude towards statistics is a multidimensional concept, and is defined as a disposition to respond favorably or unfavorably to objects, situations, or people related to statistics learning [7]. As such, it consists of affective, cognitive and behavioral components [8], [9]. A number of instruments using Likert-type responses to statements have been developed to measure attitudes towards statistics [10]. Due to the limitations of these instruments, Schau, Stevens, Dauphinee, and Del Vecchio developed a new instrument, the Survey of Attitudes Toward Statistics (SATS). The SATS consists of 36 items requiring answers on a 7-point scale, with *strongly disagree* and *strongly agree* as endpoints. The first version of SATS was designed in 1995 [8], and in 2003, it was updated by adding two more components [11].

Students’ attitudes towards statistics may play an important role in their statistics achievement, affecting the learning of statistics, understanding statistical concepts and methods, and developing useful statistical thinking skills needed to apply statistics knowledge [12], [13]. Therefore, recent studies concerning statistics courses have focused on attitudes and achievement, with growing evidence that attitudes towards statistics contribute to student success in statistical courses [14].

The SATS is a cross-cultural tool and it has been validated in different languages in previously published studies [7], . There has been no instrument adapted for Serbian students until now. The aim of this study was to investigate the psychometric properties of the Serbian version of the Survey of Attitudes Toward Statistics (SATS) in order to acquire a valid instrument to measure attitudes inside the Serbian educational context. A second aim of the present study was to explore attitudes influencing factors and the relationship between attitudes and statistics course achievement.

## Methods

The validation study was performed on a cohort of 417 medical students attending the Faculty of Medicine, University of Belgrade, who were enrolled in an obligatory introductory statistics course during the 2013–14 school year, and who were present at the statistics class on the day of the survey. The post-course version of the SATS [11] was used to measure students' attitudes towards statistics. The SATS was administered to the participants in the classroom during the last statistics class. The total time needed to complete the questionnaire ranged from 15 to 20 minutes. The response rate was 99.5%. Additional data included demographic and educational background information. Self-rating of ability in mathematics was measured by the item: How good at mathematics are you? Students were asked to rate their answers on a seven-point response scale (from 1 = very poor to 7 = very good). High scores indicated that students had high self perceptions of their overall mathematical achievements. They were also asked to fill in their current grade point averages (the mean value of grades achieved on previous exams ranged from a minimum of 6 to a maximum of 10).

Participation was voluntary and anonymous. Ethical approval for the study was obtained from the Institutional Review Board of the Faculty of Medicine, University of Belgrade.

The post-course SATS contains 36 items (SATS-36), which are grouped into six attitude components as follows: “Affect” (six items): positive and negative feelings concerning statistics, “Cognitive Competence” (six items): attitudes about intellectual knowledge and skills when applied to statistics, “Value” (nine items): attitudes regarding the usefulness, relevance, and worth of statistics in one’s personal and professional life, “Difficulty” (seven items): attitudes regarding the difficulty of statistics as a subject, “Interest” (four items): the student’s' level of individual interest in statistics and “Effort” (four items): the amount of effort a student spends on learning statistics [8], [11]. Responses for each item are ranked from 1 (strongly disagree) through 4 (neither disagree nor agree) to 7 (strongly agree), using the 7-point Likert method. According to the directions of the instrument, the answers to some negatively worded items should be reversed (1 is replaced by 7, 2 by 6, etc.). The component scores are the means of the items contained and range from 1 to 7, with higher values indicating more positive attitudes. A summary score is calculated by the mean value of all component scores.

The SATS-36 adaptation was based on internationally accepted methodology for the cultural adaptation of questionnaires. In the first step, “forward translation” (translation of the original version from English to Serbian language) was done so that the Serbian version semantically and conceptually corresponded to the original questionnaire. Translation was conducted by two independent, professional translators, one an expert in statistics and the other a professional translator. Following review and editing by translators and experts, one single translation was formed. “Backward translation” implied translation of the Serbian version of SATS-36 into English. Controversial items were discussed during the translation process, ultimately resulting in a consensus version of SATS-36 culturally adapted for Serbian students. The consensus version of the SATS-36 was administered to sixteen students to evaluate its understandability, acceptability and clarity. The final Serbian version of the SATS-36 was distributed in a validation study after expert modification of the Serbian version based on the student participant feedback.

Statistics achievement was measured using an examination form constructed by the course instructors (a written statistics test consisting of 20 multiple-choice items, with four response alternatives each; test scores ranged from 0–20). Questions related to descriptive statistics (frequency tables, measures of central tendency, measures of dispersion, types of distributions, and measures of association) and inferential statistics (basic concepts in inferential statistics, estimation, hypothesis testing). The test was administrated one week before the SATS-36 survey. Students were given 40 minutes to complete the test.

### Statistical analysis

Raw data were checked for departures from normality, and for the presence of outliers. Descriptive statistics were calculated to determine students' attitudes towards statistics. Psychometric properties of the Serbian version of the SATS-36 were analyzed through the examination of factorial structure and internal consistency (reliability). Confirmatory factor analysis (CFA) was performed to confirm the original six-dimensional structure of the SATS-36. Before running CFA, an imputation method was used to replace missing values. Box-plots were examined in order to search for univariate outliers, and values that lay more than one and a half times the length of the box from either end of the box were excluded. As a result, 23 students were eliminated from the consequent analyses, resulting in a final sample size of 392 students. Following the parceling procedure used by the authors [7], [8], items within each hypothesized subscale were grouped into parcels. Univariate distributions of the parcels were examined for the assessment of normality. Parcels’ skewness indices ranged from −0.737 to 0.124, and kurtosis indices ranged from −0.694 to 0.10. All of these indices attested that the departures from normality were acceptable. The absolute goodness-of-fit of the six-dimensional model was evaluated using the chi-square test (values that are <0.05 signify that a model may be a bad fit for the data, whereas χ^2^ values >0.05 may render the model a good fit) and three additional fit measures - Comparative-Fit Index (CFI), Tucker-Lewis index (TLI) and the Root Mean Square Error of Approximation (RMSEA). Values of CFI and TLI above 0.90 are considered adequate, whereas RMSEA values below 0.08 indicate an acceptable model fit. CFA was conducted using Amos 21 (IBM SPSS Inc., Chicago, IL, 2012). The internal consistency of the Serbian version of SATS-36 was assessed for multiple item scales by using Cronbach’s alpha coefficient (ranges from 0–1, the latter meaning perfect reliability). Multivariate linear regression analysis was used to determine factors related to students’ attitudes towards statistics. Pearson correlation coefficients were calculated to explore the relationships among the scores of the SATS-36 components and the relationships of the SATS-36 scores and statistics achievement on the test. All tests were two-tailed. P<0.05 was considered statistically significant. The IBM SPSS 21 (Chicago, IL, 2012) package was used for these analyses.

## Results

The Serbian version of the SATS-36 questionnaire was completed by 415 medical students. The mean age of the students was 21.5±0.9 years (range 20–29 years) and most participants were female (65%). Participants reported good ability in mathematics (5.10±1.55) and a current grade point average (7.96±0.86). The average statistics achievement score was 14.65±3.43.

Most of the medical students held positive attitudes towards statistics. The average total SATS score was above neutral (4.3±0.8), and varied from 1.9 to 6.2. The highest mean scores were obtained for the Cognitive Competence (4.94±1.18) and Effort components (4.88±1.20). According to the mean Affect component score (4.23±1.13), most students had positive feelings concerning statistics. The Means of Value, Interest and Difficulty components scores were close to neutral, indicating that students had neutral perceptions about the value and difficulty of statistics. Descriptive statistics of students’ attitudes towards statistics for all students, and according to gender are presented in Table 1.

**Table 1:** Descriptive statistics of student attitudes towards statistics (n = 415).

	Total (mean±sd)	Male (mean±sd)	Female (mean±sd)
**Affect**	4.23±1.13	4.23±1.04	4.23±1.18
**Cognitive**	4.94±1.18	5.02±1.11	4.91±1.22
**Value**	4.12±1.08	4.20±1.08	4.09±1.09
**Difficulty**	3.59±0.83	3.56±0.82	3.61±0.85
**Interest**	3.99±1.51	4.01±1.49	3.98±1.52
**Effort**	4.88±1.20	4.69±1.25	4.99±1.15
**Total**	4.29±0.80	4.29±0.75	4.30±0.82

**Figure 1:**
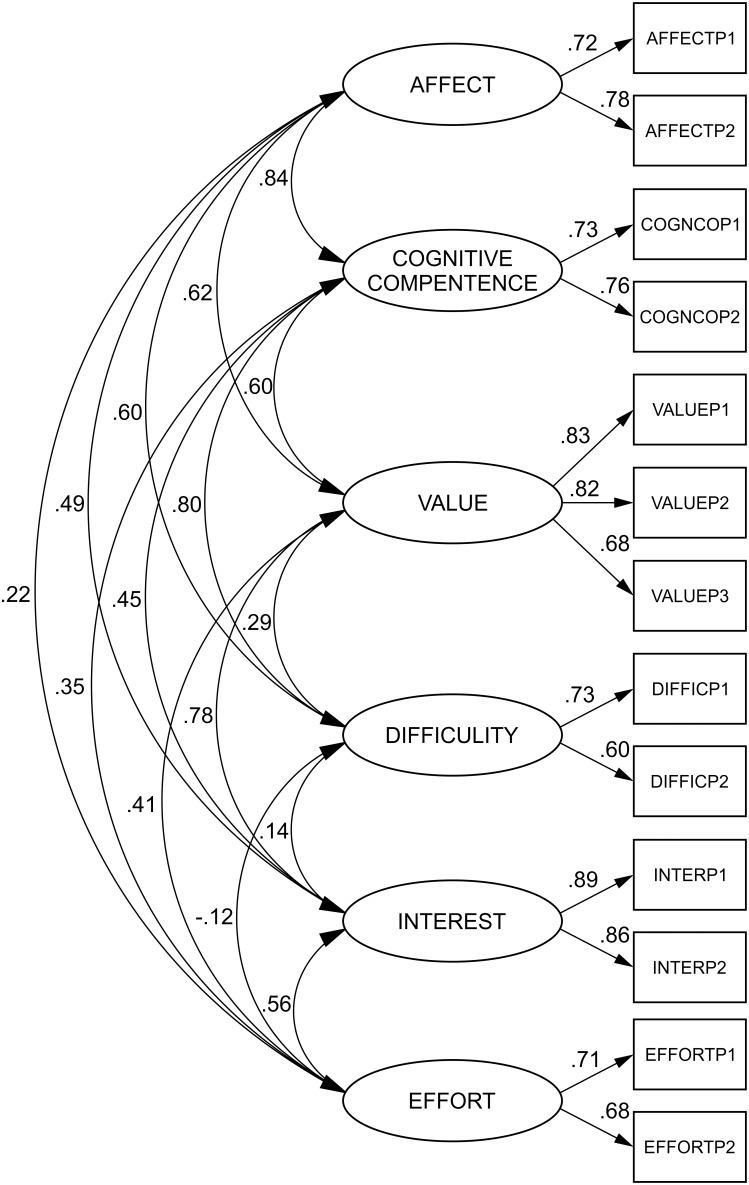
The six-factor model of the SATS 36 (standardized parameter estimates).

The six-factor structure of the SATS-36 has been validated with maximum likelihood confirmatory analysis and the results demonstrated a good fit of the data to the hypothesized six factor model. The Chi-square test rejected the six-dimensional model (χ^2^ = 130.614, df = 50, p<0.001), as we expected, due to the large sample size. The values for fit indices TLI (0.940) and CFI (0.961) were above the cut-off of ≥0.90. The RMSEA value of 0.064 (0.051–0.078) was below the suggested value of ≤0.08. In the current study, all standardized factor loadings were statistically significant and ranged from 0.60 to 0.89 (Figure 1). Correlations among factors were significant (except between Difficulty and Effort).

Analysis of internal consistency of the Serbian version of SATS-36 showed that the Cronbach’s alpha of the entire scale (items 1–36) was 0.90, indicating scale reliability. The alpha coefficients of the six components were estimated to be 0.60 for Affect, 0.75 for Cognitive Competence, 0.83 for Value, 0.57 for Difficulty, 0.88 for Interest and 0.67 for Effort.

The inter-relationships among the SATS-36 components were all positive, except between Difficulty and Effort. The Affect and the Cognitive Competence components were strongly related to each other, as well as the Interest and Value components. The Value, Difficulty and Interest components were moderately related to the Affect and Cognitive Competence components (Table 2).

**Table 2:** Correlations among the SATS components scores.

	Affect	Cognitive	Value	Difficulty	Interest
Cognitive	0.612**				
Value	0.480**	0.457**			
Difficulty	0.392**	0.525**	0.222**		
Interest	0.394**	0.364**	0.657**	0.113*	
Effort	0.166**	0.245**	0.310**	−0.079	0.439**

In a multivariate regression model, self-rating of ability in mathematics and current grade point average (GPA) were significantly associated with the total SATS-36 score after adjusting for age and gender. Students with a better self-rating of ability in mathematics and higher current GPA had higher total SATS-36 scores than those with poor self-ratings of ability in mathematics and current GPA. Except for Interest, self-rating of ability in mathematics was significantly associated with all of the other SATS-36 components after adjusting for age, gender, and current GPA. Students with better self-ratings of ability in mathematics had higher components scores than those with poor self-ratings of ability in mathematics. Gender was significantly associated with the cognitive competence SATS-36 component; males had higher cognitive competence SATS-36 score compared to females (Table 3).

**Table 3:** Regression models of variables associated with student attitudes towards statistics.

Variables	Total		Affect		Cognitive		Value		Difficulty		Interest		Effort	
	Beta	p	Beta	p	Beta	p	Beta	p	Beta	p	Beta	p	Beta	p
Age	0.047	0.338	0.013	0.794	0.017	0.700	0.025	0.629	−0.041	0.413	0.059	0.249	0.090	0.076
Sex	0.039	0.423	0.033	0.509	0.093	**0.041**	0.075	0.144	0.001	0.993	0.040	0.435	−0.089	0.081
Mathematics	0.336	**<0.001**	0.279	**<0.001**	0.486	**<0.001**	0.150	**0.004**	0.272	**0.001**	0.090	0.081	0.158	**0.002**
GPA*	0.206	**0.022**	0.125	0.174	0.141	0.089	0.183	0.052	0.064	0.498	0.221	**0.021**	0.075	0.428

*For n = 141 students; GPA = Grade Point Average.

There was a significant positive correlation between the Serbian version of SATS 36 and statistics achievement (r = 0.25). Students with more positive attitudes towards statistics tended to perform better on the test. The statistics achievement was related to the Affect (r = 0.23), Cognitive Competence (r = 0.27) and Effort (r = 0.26) components. There was no significant correlation between the Difficulty and Interest components and statistics achievement. Statistics achievement was positively correlated with self-rating of ability in mathematics and current GPA, and negatively correlated with age (Table 4).

**Table 4:** Correlations of statistics achievement and attitude components, demographic, educational background.

Variable	r	p
**Affect**	0.181	0.002
**Cognitive**	0.269	<0.001
**Value**	0.124	0.031
**Difficulty**	0.012	0.831
**Interest**	0.055	0.339
**Effort**	0.260	<0.001
**Total**	0.250	<0.001
**Age**	−0.120	0.038
**Sex**	0.001	0.988
**Mathematics**	0.207	<0.001
**GPA** *	0.207	0.030

*For n = 141 students; GPA = Grade Point Average.

## Discussion

Aiming to investigate attitudes towards statistics in medical students, a Serbian version of the SATS-36 was developed and its psychometric properties were tested. Students had no difficulty in understanding and completing the questionnaire. Means and standard deviations of the SATS-36 components were comparable to previous studies [7], [17]. The SATS-36 components’ means were neutral or above, implying positive student attitudes towards statistics.

The overall findings of the study provide evidence for the validity and reliability of the SATS-36 item version for a Serbian medical student sample. Confirmatory factor analysis validated the six-factor structure of the questionnaire (Affect, Cognitive Competence, Value, Difficulty, Interest and Effort). According to the pre-established CFI, TLI and RMSEA thresholds, the original six dimensional model can be considered as acceptable. Only the Chi-square test revealed a bad fit for the six-dimensional model analyzed. These results are not surprising considering that a major criticism against the test is that large samples tend to lead to model rejection. For the measurement model, each of the parcels loaded strongly and significantly on their hypothesized factor. The six factors were significantly correlated, with the strongest correlation emerging between Affect and Cognitive Competence. These are in accordance with previous findings from validation studies in other languages that reconfirmed the six-factor structure of the questionnaire in other students’ populations [7], [17].

The Serbian version of SATS-36 showed good internal consistency, the Cronbach’s alpha was 0.90 for the overall scale, similar to other versions [7], [17]. Internal consistency coefficients supported the reliability of each component, except for Difficulty. This finding is in accordance with those obtained in other validation studies. Schau reported that Cronbach’s alpha values ranged from 0.80 to 0.89 for Affect, from 0.77 to 0.90 for Cognitive Competence, from 0.74 to 0.91 for Value, and from 0.64 to 0.86 for Difficulty [7], [8].

In our study, self-rating of ability in mathematics was a factor influencing statistics attitudes, a finding that is consistent with previous studies [18], [19]. A recent study reported that the strongest predictor of most of the attitude components was how well medical students felt they had performed in mathematics in the past [20]. The role of sex in statistics achievement and related attitudes is controversial. A number of studies report that gender and age might influence students’ attitudes towards statistics [17], [21], [22]. Hannigan et al. reported that female students tended to score lower on all components apart from Effort compared to the male students, but there were no significant gender differences for any of the six components [20]. In our study, gender and age were not significantly associated with the SATS-36 components, except for the association among gender and cognitive competence. Our sample result is in line with the literature that reports the tendency of females to have low self-confidence in mathematics and quantitative disciplines when compared to males.

Studies investigating statistics courses analyze both cognitive and non-cognitive variables related to statistics achievement. Chiesi and Primi [23] reported that statistics achievement was related to background in mathematics, as well as to attitudes towards statistics. Significant correlations found between measures of attitudes toward statistics and statistics achievement indicated that students with positive attitudes have better test performance. This finding was consistent with the literature reporting that Cognitive Competence was most strongly correlated with statistics achievement, implying that students who felt more competent in statistics demonstrated better statistics achievement [23]–[26]. The correlation analysis also revealed that younger age, positively self-rated ability in mathematics, and higher current GPA were related to better statistics achievement.

The main limitations of the present study are the homogeneity of the participants and the fact that all of the variables were measured at a single point in time. Future longitudinal studies, including students from other academic fields and other statistics courses, are needed to further validate and generalize the present findings. The fact that the SATS-36 was administered after the test had been completed may have influenced attitudes towards statistics in the students who had good test results. For the measurement of statistics achievement, the authors did not use a standardized test (i.e. designed by course instructors), which may limit the comparisons regarding statistics achievement among published studies.

## Conclusions

The present study provided evidence for the appropriate metric properties of the Serbian version of SATS-36. Confirmatory factor analysis validated the six-factor structure of the scale. Good indices for both validity and reliability were obtained. The results reconfirmed the psychometric characteristics of the questionnaire observed in other student populations. Self-rating of ability in mathematics was a factor influencing student statistics attitudes. The significant correlations found between measures of attitudes towards statistics and statistics achievement indicate that students with positive attitudes have a better test performance. The overall finding is that SATS 36 might be a reliable and a valid instrument for identifying medical student attitudes towards statistics in the Serbian educational context.
